# The role of artificial intelligence in tackling COVID-19

**DOI:** 10.2217/fvl-2020-0130

**Published:** 2020-11-26

**Authors:** Neelima Arora, Amit K Banerjee, Mangamoori L Narasu

**Affiliations:** 1 ^1^Centre for Biotechnology, Institute of Science & Technology, JawaharLal Nehru Technological University, Hyderabad 500085, Telangana, India; 2 ^2^Biology Division, Indian Institute of Chemical Technology, Hyderabad 500007, Telangana, India

**Keywords:** 2019-nCoV, artificial intelligence, coronavirus, COVID-19, remdesivir, SARS-CoV-2, severe acute respiratory syndrome

The past two decades were marked with the outbreaks of many viral diseases such as Chikungunya, Ebola, Zika, Nipah, H7N9 Bird flu, H1N1, SARS and MERS. The world woke up to this decade with a new disease outbreak. An outbreak of a novel Coronavirus emerged in Wuhan city in the Hubei province of China in December 2019. Most of the initially identified patients were traced back to the ‘wet market’ where live animals are slaughtered and sold. The market might have played a role as an amplification hotspot from where the virus spread to other parts of China and subsequently to 213 countries and territories in a very short time. The WHO named this disease ‘COVID-19’, which is an acronym of Coronavirus Disease 2019 on 11 February 2020. As of 17 August 2020, a total of 21.2 million confirmed cases and 761,000 deaths have been reported globally [1]. The worst outbreaks of COVID-19 are reported in the USA, India, Brazil and Russia where the number of cases has surpassed the confirmed cases in China. The WHO declared the current outbreak of COVID-19 a ‘Public Health Emergency of International Concern’ on 30 January 2020 and a ‘pandemic’ on 11 March 2020.

Although the fatality rate of severe acute respiratory syndrome coronavirus 2 (SARS-CoV-2; 2.9%) is much less compared with SARS-CoV (9.6%) and MERS-CoV (34.4%), the high infectivity rate of SARS-CoV-2 compared with other coronaviruses has become a global concern. Mortality and vulnerability to COVID-19 were found to be higher in males compared with females, which could be attributed to other gendered practices such as smoking [2]. The fatality rate of COVID-19 varied with an age gradient and it was also influenced by underlying co-morbidity, in other words, conditions such as diabetes, hypertension, cancer, cardiovascular diseases and chronic respiratory disease [3–5]. Vertical transmission of COVID-19 infection from mother to baby was not observed [6]. Children are vulnerable to COVID-19 but tend to show only mild symptoms [7].

## SARS-CoV-2

The etiological agent was named as SARS-CoV-2 by the International Committee on Virus Taxonomy on 11 February 2020. SARS-CoV-2 is a beta coronavirus of zoonotic origin belonging to the subgenus *Sarbecovirus* in the *Orthocoronavirinae* subfamily of the family *Coronaviridae* transmitted to humans in a spillover event. Bats are thought to be the animal reservoir of SARS-CoV-2 but the other likely intermediate animal host is yet to be identified. The virus is a spherical particle of 70–90 nm [8], having spikes of glycoprotein projecting from its surface that bind to receptor angiotensin-converting enzyme 2 on the surface of the cell. These spikes give the virus a crown-like appearance.

The glycoprotein of SARS-CoV-2 has a furin polybasic cleavage site (PRRARS|V) located between the residues 682 and 685 at the boundary of two subunits S1/S2 that is catalyzed during biogenesis [9]. The presence of this cleavage site in SARS-CoV-2 that is observed in avian influenza viruses but not related viruses like SARS-CoV and SARSr-CoVs makes it distinct and has an impact on entry, tropism, spread and pathogenicity of the virus [9,10]. Expression of furin proteases in the respiratory tract, brain, pancreas, liver, gastrointestinal tract and reproductive organs of the host enables the virus to infect different organs and also facilitates its release into the surrounding environment in many ways. At present, 249 protein structures and 255 whole-genome sequences belonging to SARS-CoV-2 are available in the public domain.

## SARS-CoV-2 genome

A recent study suggested a single-source origin of SARS-CoV-2, as genomic sequences collected from different patients showed strikingly high identity and also indicated that SARS-CoV-2 is phylogenetically closer to bat-SL-CoVZC45 and bat-SL-CoVZXC21 [11]. Its genome size is approximately 30 kb [12]. A vast portion of the genome is occupied by two open-reading frames (ORF1a and ORF1b) that translate into pp1a and pp1ab polyproteins, which are then cleaved to 16 nonstructural proteins (nsp) like cysteine proteases, chymotrypsin-like, RNA-dependent RNA polymerase, helicase and so on. The rest of the genome encodes structural proteins like the spike(S), envelope (E), membrane (M) and nucleocapsid protein and 6–7 accessory proteins [13]. Genetic analysis revealed that SARS-CoV-2 has evolved in two lineages: ancestral S type and other more prevalent, aggressive and virulent L type derived from S type [14]. It is interesting to note that in the early stages of the epidemic, L type was more frequent, but its frequency decreased later and the frequency of S type increased, which can be attributed to differential selection pressure and epidemiological features [14].

## Transmission

COVID-19 mainly spreads from human to human through direct contact by respiratory droplets during coughing or sneezing and through indirect contact route by fomites and regularly touched surfaces [15]. SARS-CoV-2 can remain viable on various surfaces for several hours to days [16]. Air-borne transmission is possible in a medical or hospital setting in processes that generate aerosols. Although fecal–oral transmission of COVID-19 has not been reported to date, it remains a potential route [17,18].

## Clinical symptoms

Most patients experience mild flu-like symptoms including fever, cough, malaise, fatigue, sputum production and respiratory problems. Less common symptoms such as headache, hemoptysis and gastrointestinal symptoms with diarrhea and serious symptoms like pneumonia and bronchitis were also observed. Complications like Acute Respiratory Distress Syndrome, RNAaemia, acute cardiac injury, acute kidney injury and secondary infections [19] were reported in some patients. Other lab parameters associated with COVID-19 were low white blood cells and lymphocyte count, an increase in erythrocyte sedimentation rate, C-reactive protein, infiltrates and bilateral ground-glass opacity in lung CT scans.

## Prevention & control

It is imperative to adopt control measures such as case isolation, contact tracing, quarantine to limit human-to-human COVID-19 transmission. Personal hygiene measures such as frequent hand washing, respiratory hygiene, social distancing, use of face masks/shields and disinfection of surfaces can help in reducing the transmission.

## Screening & diagnosis

Discriminant clinical features like hyposmia (loss of smell) and hypogeusia (loss of taste) can be explored for preliminary diagnosis in telemedicine and mass screening [20]. Specimen samples collected from oropharyngeal and nasopharyngeal swabs or blood samples are used for diagnosis. Although routinely used for COVID-19 diagnosis in outbreak settings, sole reliance on CT scans can be misleading due to indistinguishable images with other viral pneumonia. Molecular test reverse transcriptase-PCR (RT-PCR) is recommended by WHO as the method of choice for detecting the SARS-CoV-2 nucleic acid for diagnosis of COVID-19. As the false-negative rate of RT-PCR is high, it is imperative to use CT scan of the chest as a supplementary diagnostic measure to confirm the diagnosis. Point-of-care immunodiagnostic assays that detect proteins from the COVID-19 virus or human antibodies generated against the virus in blood samples are also being used routinely to complement molecular tests due to low cost and fast results, but these methods suffer from poor sensitivity and are only qualitative [1]. Utility of these serological methods in public health settings for contact tracing and evaluating the success of nonpharmaceutical interventions has been discussed elsewhere [21]. These serological methods have now received Emergency Use Authorization by the US FDA. CRISPR-Cas12-based assay that provides rapid results can be used in point-of-care testing in the future [22].

According to recent data from WHO, 13 candidate vaccines are being evaluated. An experimental vaccine developed by the University of Oxford/AstraZeneca has entered Phase III of clinical trials while vaccine candidates from CanSino Biological Inc./Beijing Institute of Biotechnology and Moderna/NIAID have reached Phase II trials and ten vaccine candidates have reached Phase I/II and Phase I stages. 129 other candidate vaccines are in the preclinical stage (WHO) and many are in pipeline [23,24].

## Therapeutic agents

Some of the potential drugs against COVID-19 beingconsidered and evaluated are remdesivir (GS-5734), baricitinib, a combination drug ritonavir/lopinavir, Ribavirin^®^, umifenovir and IFN-β and other broad spectrum antiviral agents. Remdesivir was not found to be effective in treating COVID-19 patients in a placebo-controlled randomized trial of remdesivir [25]. In a recent development, the FDA has approved the use of remdesivir in confirmed and suspected cases of COVID-19. As of 25 June 2020, about 1235 clinical trials for various therapeutic agents against COVID-19 are being conducted across the globe [26].

## Application of artificial intelligence in COVID-19 disease management

Unprecedented pace of efforts to address the COVID-19 pandemic situation is leveraged by big data and artificial intelligence (AI). Various offshoots of AI have been used in several disease outbreaks earlier. AI can play a vital role in the fight against COVID-19.

AI is being successfully used in the identification of disease clusters, monitoring of cases, prediction of the future outbreaks, mortality risk, diagnosis of COVID-19, disease management by resource allocation, facilitating training, record maintenance and pattern recognition for studying the disease trend. Several applications of AI that are garnering a lot of interest and raising hopes in the fight against COVID-19 are as follows:

### AI in prediction & tracking

AI can be harnessed for forecasting the spread of virus and developing early warning systems by extracting information from social media platforms, calls and news sites and provide useful information about the vulnerable regions and for prediction of morbidity and mortality. Bluedot identified a cluster of pneumonia cases and predicted the outbreak and geographical location of the COVID-19 outbreak based on available data using machine learning. HealthMap collects the publicly available data on COVID-19 and makes it readily available to facilitate the effective tracking of its spread. Recently, the role of AI in identification and forecasting of COVID-19 outbreaks by employing multitudinal and multimodal data was emphasized [27].

### AI in contact tracing

AI can augment mobile heath applications where smart devices like watches, mobile phones, cameras and range of wearable device can be employed for diagnosis, contact tracing and efficient monitoring in COVID-19 [28]. Applications like AI4COVID-19 that rely on audio recording samples of 2 s cough can be used in telemedicine [29].

### AI in monitoring of COVID-19 cases

AI techniques are applied for monitoring patients in clinical settings and prediction of course of treatment. Based on the data derived from vital statistics and clinical parameters, AI may provide critical information for resource allocation and decision-making by prioritizing the need of ventilators and respiratory supports in the Intensive Care Unit [30]. AI can also be used for predicting the chances of recovery or mortality in COVID-19 and to provide daily updates, storage and trend analysis and charting the course of treatment.

### AI in early diagnosis

AI was used for the detection and quantification of COVID-19 cases from chest x-ray and CT scan images [31–33]. Researchers have developed a deep learning model called COVID-19 detection neural network (COVNet), for differentiating between COVID-19 and community-acquired pneumonia based on visual 2D and 3D features extracted from volumetric chest CT scan [34]. Singh *et al.* developed a novel deep learning model using Multi-Objective Differential Evolution and convolutional neural networks for COVID-19 diagnosis using a chest CT scan [35]. COVID-ResNet developed using automatic and discriminative learning rate and progressive image resizing performed better than COVID-Net in diagnosing COVID-19 [36]. Alom *et al.* developed a system called COVID_MTNet by applying improved Inception Recurrent Residual Neural Network and NABLA-3 network models for detection and localization of regions of interests from both x-ray images and chest CT scan [37]. Another study used AI-based classifiers for predicting the outcome of RT-PCR results of COVID-19 cases using 16 simple parameters derived from complete blood profile [38]. This may find application in reducing the number of RT-PCR tests in resource-poor settings.

### AI in reducing the burden from medical practitioners & healthcare staff

AI-based triage systems can help in reducing the work burden of medical staff and healthcare workers by automating several processes such as imparting training to practitioners, determination of the mode of treatment and care by analyzing clinical data using pattern recognition approaches, digitalization of patient’s reports and also by offering solutions that minimize their contact with the patients [39–41]. AI can be used for classification of patients based on the severity of symptoms, genetic disposition and clinical reports in different categories like mild, moderate and severe, so that different approaches can be adopted for handling the patients in the most effective manner. AI in telemedicine can also be used to eliminate the need of frequent and unnecessary hospital visits by distant monitoring of cases and recording of patient’s data in asymptomatic cases or patients with mild symptoms. AI-based medical chatbots can also be used for consultations, thereby reducing the physical crowding of hospitals as well as the spread of infection and thus prevent weighing down of efficient operation of critical care services [42,43]. Chatbots like Clara from the Centre for Disease Control and Zini are providing much needed support to patients in remote settings [44]. A prognostic prediction algorithm predicted the mortality risk of patients by machine learning methods using extracted features derived from the data of other patients as training dataset [45]. A similar approach was used to predict the possibility of developing acute respiratory distress syndrome [46]. Service robots and anthropomorphic robots with AI core can be used for the delivery of essential services and routine tasks like cleaning, disinfecting and monitoring in hospital settings [47,48].

### AI in protein structure prediction

AI can help in predicting the structure of important proteins crucial for virus entry and replication and provide useful insight that can pave way for drug development in a very short time. AlphaFold algorithm of Google Deep mind employed deep residual networks (DRN) called ResNets for predicting protein structures of membrane protein, protein 3a, nsp2, nsp4, nsp6 and papain-like C-terminal domain of SARS-CoV-2, which will give huge impetus to drug discovery programs [49]. DeepTracer, a program based on customized deep convolutional neural network, was used to derive protein complex structure of SARS-CoV-2 from high-resolution cryoelectron microscopy density maps and amino acid sequence [50].

### AI in development of therapeutics

AI techniques can boost and complement traditional technologies by reducing the time required in bringing a drug from bench to bed by speeding up lead discovery, virtual screening and validation processes by a huge margin. AI can also accelerate the pace by deriving useful data for drug repurposing or drug repositioning by screening properties of already approved and validated drugs based on molecular descriptors and properties, which may not be possible for a human expert. BenevolentAI used machine learning methods to accelerate its drug discovery program and identified baricitinib as a potential drug against COVID-19 [51,52]. Insilico Medicine has identified several small molecules against COVID-19 using AI [53]. Another study combined virtual screening and supervised learning to identify potential drugs against COVID-19 [54]. Zhou *et al.* adopted an integrative network-based systems pharmacological methodology for finding potential drugs for SARS-CoV-2 from the already existing repertoire of drug molecules and drug combinations [53]. Several other AI-based endeavors including inclProject IDentif.AI (identifying infectious disease combination therapy with artificial intelligence) [55] and PolypharmDB [56] have been successful in identifying candidates against COVID-19. Many machine learning approaches and deep learning-based applications are also being used for expediting the drug discovery process [57–60].

### AI in development of vaccines

Never before has mankind witnessed such a race for the development of a vaccine against a pathogen. The pace of the discovery can be accelerated manifold by harnessing the power of AI. Ong *et al.* predicted possible vaccine candidates for COVID-19 using the Vaxign reverse vaccinology-machine learning platform that relied on supervised classification models [61].

### AI in curbing spread of misinformation

Due to the avalanche of information, this pandemic has turned into an infodemic. Understanding knowledge, awareness and practices toward COVID-19 by tapping information from social media platforms like Twitter, Facebook etc. can help in devising the strategy to assemble and disseminate timely and correct information for mitigating the impact of COVID-19 [62,63]. Machine learning techniques can be used to identify trends and sentiment analysis and provide information regarding the origin of false information and help in curtailing the rumors and misinformation [64]. AI techniques can further be used for presenting a clear picture of recovery rates, accessibility and availability to healthcare and identification of the gaps. AI can provide the latest updates about the emerging evidence in diagnosis, treatment, spectrum of symptoms and therapeutic outcomes in this highly dynamic situation, which will help clinicians in real-world scenario and help public in overcoming fear and panic [65].

### AI in genomics

Randhawa *et al.* devised a method for fast and accurate classification of available SARS-CoV-2 genomes by applying machine learning on identified genomic signatures [51]. Wang *et al.* used ontology-based side effect prediction framework and Artificial Neural Network to evaluate the side effects of Traditional Chinese Medicines for the treatment of SARS-CoV-2 [66].

## Conclusion & future perspective

Adopting a three-pronged approach based on testing, isolation and contact tracing is warranted to combat COVID-19. It is necessary to exploit the available knowledge base to develop effective chemotherapeutic agents against COVID-19, taking cues from lessons learnt in the past during other such outbreaks.

As there is no silver bullet available to cure the disease, we need to hasten progress on all fronts ranging from surveillance and monitoring to prevention and treatment. As this is the third outbreak of a coronavirus in recent times and many coronaviruses are circulating in animal reservoirs, we must focus on deciphering the molecular mechanism of SARS-CoV-2 and other coronaviruses and increasing our preparedness by capacity building for preventing future outbreaks [67]. As the current scenario warrants the need for immediate delivery of solutions, response to this outbreak was hugely augmented by various digital technologies and AI [68]. AI was found to be on par with and even more accurate than human experts in COVID-19 diagnosis and drug discovery. We need bigger datasets for training AI models and a legal framework and ethical considerations for sharing data before AI takes the forefront in diagnosis and other areas. Several bottlenecks in harnessing AI to its full potential in the current scenario are availability and sharing of clinical and epidemiological data, computational resources, scalability, privacy and ethical concerns.
